# Production, biodistribution assessment and dosimetric evaluation of ^177^Lu-TTHMP as an agent for bone pain palliation

**Published:** 2015

**Authors:** Samaneh Zolghadri, Hassan Yousefnia, Amir Reza Jalilian, Mohammad Ghannadi-Maragheh

**Affiliations:** Nuclear Science and Technology Research Institute (NSTRI), Tehran, Iran

**Keywords:** Bone pain palliation, Dosimetry, Lu-177, *TTHMP*

## Abstract

**Objective(s):**

Recently, bone-avid radiopharmaceuticals have been shown to have potential benefits for the treatment of widespread bone metastases. Although ^177^Lu-triethylene tetramine hexa methylene phosphonic acid (abbreviated as ^177^Lu-TTHMP), as an agent for bone pain palliation, has been evaluated in previous studies, there are large discrepancies between the obtained results. In this study, production, quality control, biodistribution, and dose evaluation of ^177^Lu-TTHMP have been investigated and compared with the previously reported data.

**Methods:**

TTHMP was synthesized and characterized, using spectroscopic methods. Radiochemical purity of the ^177^Lu-TTHMP complex was determined using instant thin-layer chromatography (ITLC) and high performance liquid chromatography (HPLC) methods. The complex was injected to wild-type rats and biodistribution was studied for 7 days. Preliminary dose evaluation was investigated based on biodistribution data in rats.

**Results:**

^177^Lu was prepared with 2.6-3 GBq/mg specific activity and radionuclide purity of 99.98%. ^177^Lu-TTHMP was successfully prepared with high radiochemical purity (>99%). The complex showed rapid bone uptake, while accumulation in other organs was insignificant. Dosimetric results showed that all tissues received almost insignificant absorbed doses in comparison with bone tissues.

**Conclusion:**

Based on the obtained results, this radiopharmaceutical can be a good candidate for bone pain palliation therapy in skeletal metastases.

## Introduction

Bone metastasis is a common and severe complication in advanced stages of cancer (1, 2). It develops in up to 70% of patients with prostate cancer and breast cancer, and in nearly 30% of those with lung, bladder, and thyroid cancers (3, 4).

Standard treatments for this condition include systemic therapies (e.g., analgesics, chemotherapy, hormonal therapy, and bis-phosphonates) and local control (e.g., radiation therapy, radiofrequency ablation, and surgical stabilization of the affected site) (5).

The proven efficacy and minimal toxicity of radiation therapy make it very suitable for pain palliation in cancer patients (3). Radionuclide therapy, by using specific tumor-seeking radiopharmaceuticals, can be applied as a treatment for bone metastases. Recently, various phosphonate ligands, labeled with β-emitting radionuclides, have been effective in metastatic bone pain palliation (6, 7).

Successful bone pain palliation is based on selective concentration and prolonged retention of the radiopharmaceutical at skeletal lesions, while keeping the bone marrow absorbed dose as low as possible (8); for this purpose, low energy β-particles are recommended.

Recently, radiopharmaceuticals with radio-nuclides such as ^32^P, ^89^Sr, ^186^Re, ^188^Re, ^153^Sm, ^166^Ho, and ^177^Lu have been developed for the treatment of painful metastases. Among these radioisotopes, ^177^Lu [t_1/2_=6.73 d, E_β_(max) = 497 keV, Eγ=112 keV (6.4%), 208 keV (11%)] has notable characteristics. The significant advantage of utilizing ^177^Lu is its β-particle energies which are adequately low; therefore, bone marrow suppression is minimum when it accumulates in skeletal lesions (9, 10). Various bone-avid agents with ^177^Lu have been developed and used for pain palliation including ^177^Lu-DOTMP (11) and ^177^Lu-EDTMP (12).

Improved cancer treatments and increased emphasis on the quality of life of cancer patients have led to a search for effective pain palliation for clinically active bone metastases, with fewer short-term and long-term side-effects. Therefore, various ^177^Lu complexes including PYP (13), DTPMP, TTHMP (14), MDP (15), HEDP (16), and CTMP (17) have been developed and evaluated.

Among these radiopharmaceuticals, ^177^Lu-triethylene tetramine hexa methylene phosphonic acid (abbreviated as ^177^Lu-TTHMP) has been shown to have favorable characteristics. This complex accumulates massively in the bone, while its accumulation in other critical organs is very slow. Previous studies on ^177^Lu-TTHMP have shown that this complex is comparable to other radiopharmaceutical complexes such as ^188^Re-HEDP and ^177^Lu-EDTMP, used in clinical trials; however, the results of these studies are inconsistent. Furthermore, despite the direct relationship between the absorbed dose and the effect of radiopharmaceuticals in disease management, the absorbed dose of this complex has not been reported, so far.

**Figure 1:**
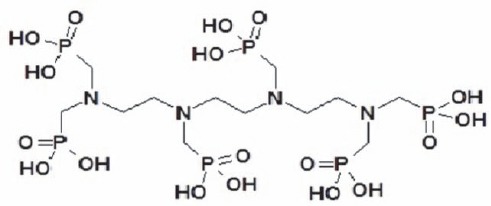
The chemical structure of TTHMP

Accordingly, in this study, TTHMP was synthetized and labeled with ^177^Lu. The chemical structure of the ligand is demonstrated in Figure 1. The parameters affecting radiochemical purity such as ligand concentration, pH of the reaction mixture, and incubation time were investigated. Biodistribution of the complex in wild-type rats was surveyed for better comparison with the reported studies. Finally, the preliminary dose evaluation in different human organs was investigated based on distribution data in rats.

## Methods

^177^Lu was produced by irradiation of natural Lu_2_O_3_ target at a thermal neutron flux of approximately 4×10^13^ n/cm^2^/s for 5 days at Tehran Research Reactor (TRR). Whatman No. 3 paper was obtained from Whatman, UK.

Radiochromatography was performed using a Bioscan AR-2000 radio-TLC scanner (Bioscan, France). Analytical high performance liquid chromatography (HPLC) method was applied to determine specific activity of the complex by a Shimadzu LC-10AT, equipped with two detector systems, flow scintillation analyzer (Packard-150 TR), and UV-visible spectrophotometer (Shimadzu), using Whatman Partisphere C-18 column, 250×4.6 mm (Whatman Co., NJ, USA).

A high-purity germanium (HPGe) detector, coupled with a Canberra™ multichannel analyzer (model GC1020-7500SL, Canberra Industries Inc., CT, U.S.A.) and a dose calibrator ISOMED 1010 (Elimpex-Medizintechnik, Austria) was used for measuring the distributed activity in rat organs. All other chemical reagents were purchased from Merck, Germany.

Calculations were based on the 112 kev peak for ^177^Lu. All values were expressed as mean ± standard deviation, and the data were compared using Student’s T-test. Animal studies were carried out in accordance with the United Kingdom Biological Council’s Guidelines on the Use of Living Animals in Scientific Investigations (2^nd^ edition). The approval of NSTRI Ethical Committee was obtained for conducting this research.

The wild-type rats (NMRI), weighing 180-200g, were purchased from Pasteur Institute of Karaj, Iran, and were acclimatized to proper rodent diet.

### Production and quality control of ^177^LuCl_3_ solution

Lutetium-177 was produced by neutron irradiation of 1 mg of natural Lu_2_O_3_ (99.999% from Aldrich Co., UK), according to the reported procedures (18) at Tehran Research Reactor (TRR). The irradiated target was dissolved in 200 µL of 1.0 M hydrochloric acid (HCl) to prepare ^177^LuCl_3_; then, it was diluted to the appropriate volume with ultrapure water to produce a stock solution with the final volume of 5 ml (0.04 mol/l).

The mixture was filtered through a 0.22 µm filter for sterilization (Waters, U.S.A). The radionuclidic purity of the solution was tested for the presence of other radionuclides, using HPGe spectroscopy for the detection of various interfering gamma-emitting radionuclides. The radiochemical purity of ^177^LuCl_3_ was checked using 2-solvent systems for instant thin-layer chromatography (ITLC) [A: 10 mmol/l diethylene triamine pentaacetic acid (DTPA) at pH 5 and B: 10% ammonium acetate-methanol (1:1)].

### Synthesis of TTHMP

The experimental procedure for the synthesis of TTHMP ligand was in accordance with other bisphosphonates, as reported (14). Briefly, a quantity of 0.48 g (3.3 mM) of triethylenetetramine was dissolved in 0.75 ml of concentrated HCl and a concentrated aqueous solution of 1.62 g phosphorous acid (20 mM). The resulting solution was heated to the reflux temperature and 3.2 ml of 37% aqueous formaldehyde solution (40 mM) was added dropwise during 1 h to the refluxing solution, and the refluxing was continued for another 1 h. The result of the reaction was a precipitated ethanol of a white product from the concentrated reaction solution.

### Radio-labeling of TTHMP with ^177^LuCl_3_

A stock solution of TTHMP was prepared by dissolution in 1 N NaOH) and was diluted to the appropriate volume with ultrapure water through dissolving a specific amount of ligand in 1.5 ml NaOH (2 N) and 3.5 ml distilled H_2_O (pH=12). Then, 0.3 ml of this solution was added to 200 µl ^177^LuCl_3_ (210.9 MBq) and pH was adjusted to 7, using a phosphate buffer.

The reaction mixtures were incubated by stirring at room temperature for 1 h. Various parameters such as ligand concentration, pH of the reaction mixture, and incubation time were optimized to achieve maximum complexation yield. The radiolabeling yield of the ligand was determined with paper chromatography, using Whatman No. 3 paper by sampling 5 µl of the reaction mixture on the paper strip, developed in NH_4_OH:MeOH:H_2_O (1:10:20) mixture.

### Stability studies

Stability of the complexes stored at room temperature (22°C) and in human serum (37°C) was studied at different intervals by determining the radiochemical purity of complexes, using paper chromatography in NH_4_OH:MeOH:H_2_O (1:10:20) system.

### In vitro protein binding of ^177^Lu-TTHMP in presence of human serum

In vitro protein binding of the complex was carried out in human blood by protein precipitation. One ml of the labeled complex was mixed with 3 ml of fresh human plasma, and incubated for 1 h at 37°C. Content of the tube was centrifuged at 3000 rpm for 10 min in order to separate the serum from blood cells. After mixing an approximately equal volume of 10% trichloroacetic acid (TCA), the mixture was centrifuged at 3000 rpm for 10 min. Residue was separated from supernatant and both layers were counted for radioactivity in a well-type gamma counter. Protein binding of the complex was expressed as a fraction of protein-bound radioactivity in percentage of total radioactivity.

### Biodistribution of ^177^Lu-TTHMP in wild-type rats

Final ^177^Lu-TTHMP solution of 3.7 MBq in 50-100 μl was injected intravenously to the rats through their tail vein. The animals were sacrificed at specified time intervals (2, 4, 24, 48, 72, and 168 h), and specific activity of different organs was calculated as the percentage of injected dose per gram (%ID/g), using HPGe detector.

### Dosimetric studies

The absorbed dose of each human organ was calculated by medical internal radiation dosimetry (MIRD) method, based on biodistribution data in wild-type rats. For this purpose, first the accumulated source activity was calculated by plotting the clearance curves for each organ and computing the area under the curves. Then, the accumulated activity in animals was extrapolated to the accumulated activity in humans by the proposed method of Sparks et al. (equation 1) (19).


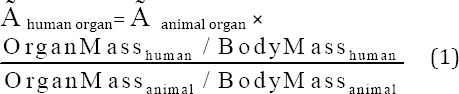


In order to extrapolate the accumulated activity to human, the mean weight of each organ for standard human were considered. Finally, the absorbed radiation dose was calculated by MIRD formulation (20):


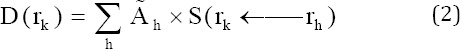


Where D(r_k_) is the absorbed dose of the target organ and S(r_k_) is defined as the mean absorbed dose to the target region r_k_ per unit accumulated activity in the source region r_h_. The S factors have been obtained from OLINDA software (21)

## Results

### Radionuclide production

The radionuclide was prepared in the range of 2.6-3 GBq/mg specific activity for radiolabeling use. After counting the samples on the HPGe detector for 5 h, two major photons (6.4% of 0.112 MeV and 11% of 0.208 MeV) were observed.

The radiochemical purity of the ^177^Lu solution was checked in the 2 solvents. In 10 mmol/l DTPA aqueous solution (solvent 1), free Lu^3+^ cation was complexed to more lipophilic Lu-DTPA form and migrated to higher R_f_. The small radioactive fraction, which remained at the origin, could be related to other Lu ionic species, which were not involved in forming Lu-DTPA complex, such as LuCl_4_^-^ and/or colloids. On the other hand, 10% ammonium acetate-methanol mixture (1:1) (solvent 2) was used for the determination of radiochemical purity.

### Ligand synthesis

The TTHMP ligand was synthesized and its structure was determined using Proton Nuclear Magnetic Resonance (^1^H NMR), mass, and infrared (IR) methods.

TTHMP: [m.p. 90-92°C, ^1^H-NMR (D_2_O, δ [ppm]): 3.02-3.25(m, 12 H, >N-CH_2_CH_2_-N<), 3.37-3.47(m, 12 H, -NCH_2_-PO_3_H_2_)].

Mass: m/z (%), 710 (M+, % 26), 370 (%55), 233 (%44), 116 (%63), 110 (%100).

### Labeling optimization studies

**Figure 2:**
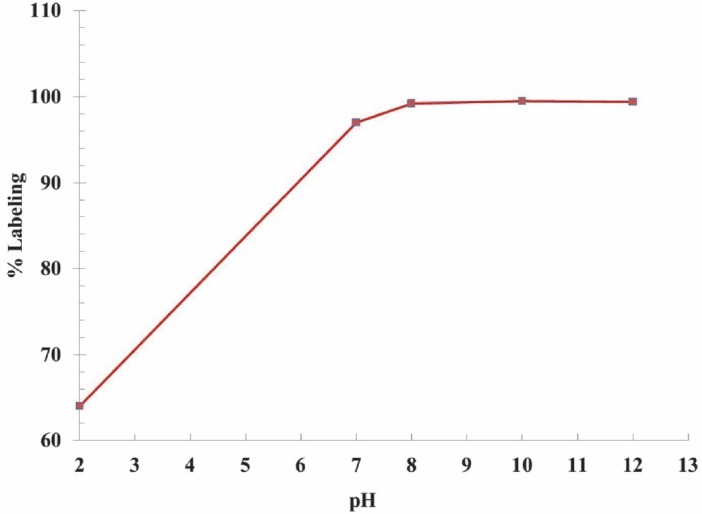
Effect of pH variation on complexation yield of ^177^Lu-TTHMP at room temperature

In order to obtain maximum complexation yields, several experiments were carried out by various reaction parameters such as ligand concentration, pH, and reaction time. The effect of pH variation on complexation yield of all ligands at room temperature was also studied by varying the pH of reaction mixture from 2 to 12, using 1 M HCl or 2 M NaOH solution. Maximum yield of 99% was observed at pH=7-8 for complexes. The effect of pH on complexation yield is shown in Figure 2.

Ligand concentration was varied within a wide range of 10 to 50 mg/ml for each ligand. It was observed that at room temperature, complexation >99% was achieved with 50:1 ligand-molar ratio.

**Figure 3:**
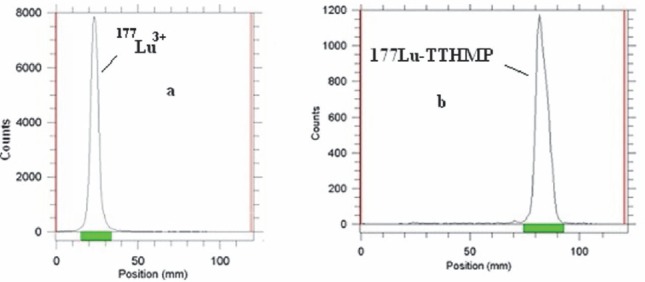
(a) ITLC chromatograms of ^177^LuCl_3_ solution, and (b) ^177^Lu-TTHMP in a NH_4_OH: MeOH: H_2_O (0.2:2:4) solution, using Whatman 3 MM

The reaction mixture was incubated at room temperature for different time periods and 60 min incubation was found to be adequate to

yield maximum complexation. The best ITLC mobile phase was Whatman 3 MM paper, using NH_4_OH: MeOH: H_2_O (1:10:20), as shown in Figure 3.

Although the ITLC studies confirmed the production of the radiolabeled compound, HPLC studies demonstrated the existence of one radiolabeled species, utilizing both UV and scintillation detectors.

### Stability studies

The stability of ^177^Lu-TTHMP, prepared under optimized reaction conditions, was studied, and the complex showed excellent stability both in human serum at 37°C and at room temperature after 72 h.

In vitro protein binding of the complex was carried out in human blood by protein precipitation. Protein binding of the complex was expressed as a fraction of protein-bound radioactivity in percentage of total radioactivity. The complex protein binding was approximately 58–60%.

### Biodistribution of ^177^Lu-TTHMP in wild-type rats

**Figure 4:**
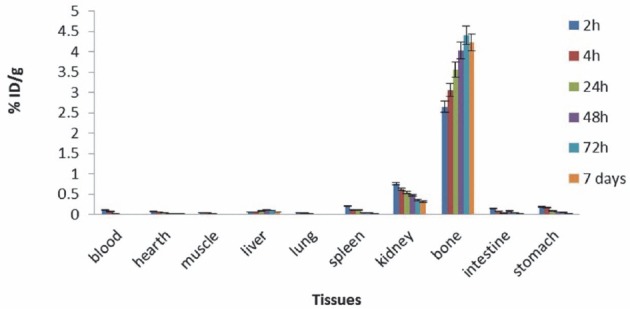
Biodistribution of ^177^Lu-TTHMP in different organs of wild-type rats

The tissue uptake of the complex was calculated as the percentage of area under the curve of the related photopeak per gram of tissue (% ID/g) (Figure 4). The major radioactivity was accumulated in bones, as expected for bone-avid radiopharmaceuticals; the complex was also excreted through the kidneys.

### Dosimetric studies

**Figure 5:**
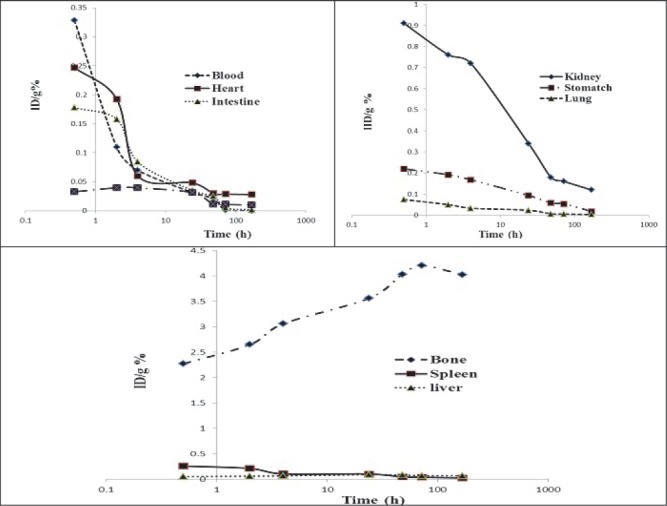
The clearance curves of each organ of rats

Preliminary dosimetric evaluation in human organs was performed by MIRD method, based on biodistribution data in rat organs. First, the area under the clearance curve was calculated for each organ (Figure 5). Then, the absorbed dose was calculated according to the s factors in OLINDA software. The absorbed doses in each organ after ^177^Lu-TTHMP injection are shown in Table 1.

**Table 1:** Absorbed dose in each organ after the injection of ^177^Lu-TTHMP

Organ	Absorbed dose (mSv/MBq)	Organ	Absorbed dose (mSv/MBq)
Adrenal glands	0.032	Ovaries	0.019
Brain	0.036	Pancreas	0.020
Breasts	0.009	Red Mar.	2.538
GB Cont.	0.013	Cort Bone Sur.	6.049
LLI Cont.	0.035	Trab. Bone Sur.	7.731
SI Cont.	0.017	Cort Bone Vol.	1.441
Stom. Cont.	0.018	Trab. Bone Vol.	3.716
ULI Cont.	0.014	Spleen	0.031
Heart Cont.	0.023	Testes	0.011
Heart Wall	0.034	Thymus	0.014
Kidneys	0.114	Thyroid	0.023
Liver	0.048	UB Cont	0.011
Lungs	0.022	Uterus	0.014
Muscle	0.028	Tot. Body	0.496

## Discussion

The radiolabeled ^177^Lu-TTHMP was prepared with high radiochemical purity (>99%, ITLC) and by specific activity of 2.6-3 GBq/mg. ^177^Lu-TTHMP demonstrated a great stability at room temperature. The final product was administered to wild-type rats and biodistribution of the radiopharmaceutical was checked 2-168 h later. The results showed massive accumulation in the bone tissue, which increased up to 7 days (4.22 %ID/g). The complex was rapidly cleared of blood and no significant uptake was observed in critical organs. 20^177^Lu-TTHMP has been prepared and reported in previous studies (14, 16). In these studies, significant accumulation was reported in the bone, while accumulation in other organs was negligible. However, in this study, the complex pharmacokinetics in the bone followed a pattern similar to previously reported research, although the amount of radioactivity in the bone (%ID/g) was considerably different.

Lungu et al. investigated the biodistribution of ^177^Lu-TTHMP in rats, demonstrating a rapid uptake in bone 4 h after the injection (79 %ID/g). Accumulation in the bone increased until 72 h after the injection and reached about 93 %ID/g. In the study by Chakraborty et al., the bone uptake was reported as 6.58 %ID/g, 3 h, which increased up to 7.82 %ID/g 96 h after the injection. In the current study, the maximum bone uptake of 4.40 %ID/g was obtained 72 h after the injection, which is in agreement with the findings of Chakraborty and colleagues (14).

According to Mitterhauser’s findings (22), radiopharmaceutical preparation conditions strongly influence bone binding. Therefore, the differences in bone accumulation of ^177^Lu-TTHMP, reported in various studies, may be due to variations in preparation conditions of the complex. The optimum conditions for radiolabeling of the complex in this research and previous studies are provided in Table 2.

**Table 2:** Optimum conditions for radiolabeling of ^177^Lu-TTHMP

Reference	The current study		Ref. 14	Ref. 16
**Ligand/metal**	50:1	60:1		-
**pH**	7-8	9		7-7.5
**Temperature**	Room temperature	Room temperature		90°C
**Time**	60 min	15 min		90 min
**Radiochemical purity**	>99%	97.7%		>98%

In addition to the bone, low accumulations were observed in the liver, kidney, and spleen. The amount of radioactivity (%ID/g) in these tissues is demonstrated in Table 3. According to the results of Chakraborty et al., maximum liver uptake (0.15 %ID/g) was reported 3 h after the injection, while the best liver uptake was obtained after 48 h in the current study (0.10 %ID/g). Except the liver uptake, the pharmacokinetics of the complex in kidney and spleen followed a similar pattern, demonstrating the uptake reduction of kidney and spleen with time.

In this study, the absorbed dose of the complex was investigated for the first time. As expected, the highest absorbed dose for this complex was observed in the bone surface with 7.731 mSv/MBq.

**Table 3:** Comparison of liver, kidney, and spleen uptake for ^177^Lu-TTHMP complex in normal rats (%ID/g)

	2 h	3 h	4 h	24 h	48 h	References
**Liver**	0.06	-	0.06	0.09	0.10	This study
	-	0.15	-	0.13	0.13	Ref. 14
**Kidney**	0.76	-	0.62	0.54	0.48	This study
	-	0.48	-	0.37	0.36	Ref. 14
**Spleen**	0.21	-	0.11	0.11	0.04	This study
	-	0.38	-	0.19	0.26	Ref. 14

## Conclusion

According to the results, ^177^Lu-TTHMP massively accumulated in the bone, while no significant accumulation was observed in other organs. Also, the dosimetric results demonstrated that the bone surface/red marrow dose ratio was approximately 3, while this ratio was 1.5 for ^166^Ho-DOTMP (^6^). Considering these desirable characteristics, this radiopharmaceutical can be a good candidate for bone pain palliation therapy in skeletal metastases.
